# Dataset of costs of the mitigation hierarchy and plant translocations in France

**DOI:** 10.1016/j.dib.2021.107722

**Published:** 2021-12-16

**Authors:** Margaux Julien, Bruno Colas, Serge Muller, Bertrand Schatz

**Affiliations:** a CEFE, CNRS, Univ Montpellier, EPHE, IRD, Montpellier, France; b Ecotonia, 60 rue tourmaline, Eguilles 13510, France; c Université Paris-Saclay, CNRS, AgroParisTech, Ecologie Systématique et Evolution, Orsay 91405, France; d Muséum National d'Histoire Naturelle, UMR 7205, MNHN-CNRS-UPMC-EPHE-UA, CP 39, 16 rue Buffon, Paris 75005, France

**Keywords:** Environmental impact assessment, Offsets, Land planning, Mitigation translocation

## Abstract

These data are coming from the derogation requests for the destruction of protected species in the context of construction or development work in France. These derogation requests include, among other things, the contents of an environmental impact assessment and the costs of the measures suggested to reduce the impact on the environment. In the article connected to this dataset, we studied the quality of the plant translocation protocols proposed in 95 derogation files (see Julien et al., 2022). We additionally collected during the reading of the files data that we make available here about the costs of (i) the total project, (ii) the mitigation hierarchy and (iii) the plant translocation operations and monitoring. These data complement our aforementioned paper by documenting how much translocations cost and in what proportion of the other costs reported in the projects we evaluated for quality. These data can be helpful for environmental stakeholders but also to further studies to determine the extent to which the environment is considered in land planning.

## Specifications Table

**Table:** 

Subject	Environmental Science – Management, Monitoring, Policy and Law
Specific subject area	Economic costs, mitigation hierarchy, plant translocation
Type of data	Table
How the data were acquired	The data is extracted from the derogation request files without modifications. These files are requested by the French administration when protected species are impacted by a development project and include the information contained in an environmental impact assessment.
Data format	Raw
Parameters for data collection	We have collected data present in the derogation files made in France between 2018 and 2020. These data led to an article on the quality of plant translocations in derogation files. In addition to this article, we present here other data collected on the same occasion, focusing on costs. They complement the analysis on translocation protocols, giving the projected costs of translocations and the costs of the mitigation hierarchy and the total project.
Description of data collection	For each file, different parameters were determined when reading them. In particular, the total cost of the project and the mitigation hierarchy cost are noted. It is important to know these costs, as it shows how much money is dedicated to reducing environmental impacts in a development project. The original article focuses on plant translocations, so we also recorded the costs of translocation operations. An operation may concern one or more species. The cost of the operation, the cost of the monitoring, and the cost of the whole operation are recorded for each file. The translocation operations presented here are the same as Julien et al. [1].
Data source location	Not applicable
Data accessibility	Repository name: Data costs of land planning projects and plant translocations Data identification number: doi: 10.17632/k72c3pfr9r.1 Direct URL to data: https://data.mendeley.com/datasets/k72c3pfr9r/1
Related research article	M. Julien, B. Colas, S. Muller, B. Schatz, Quality assessment of mitigation translocation protocols for protected plants in France, J. Environ. Manag. 302, 114064 (2022) https://doi.org/10.1016/j.jenvman.2021.114064

## Value of the Data

• These data are helpful as it gives a better idea of the proportion of the mitigation hierarchy within a project. These data are not easily accessible, so it is difficult to know these costs.
• The mitigation hierarchy costs can indicate the extent to which the environment is considered in development projects.
• These data can be used by (1) environment stakeholders to better estimate the costs of mitigation hierarchy of a new project, and (2) the French administration to help them make decisions on environmental policies.
• These data only cover 2018 to 2020 and may complement future investigations on the subject.

## 1 Data Description

The file “Data costs.xlsx” provides information on the costs of the mitigation hierarchy and the total cost of a development project. Among the 92 files studied, the cost of the entire mitigation hierarchy was indicated in 80.4% of the files and only in 38.0% of the files (N=92) for the total project cost. The median cost of the mitigation hierarchy was about 399,595 € (median value calculated on 74 files; 1st quartile = 126,087 €; 3rd quartile = 913,925 €), while the median total project cost was about 20,000,000 € (median value calculated on 35 files; 1st quartile = 7,906,357 €; 3rd quartile = 50,500,000 €). The mitigation hierarchy represented 1.96% of total project costs (median value calculated on 28 files; 1st quartile = 0.53%; 3rd quartile = 4.96%).

The file “Data costs translocation.xlsx” provides information on translocation cost, which was indicated in 63.1% of cases, and 48.5% of cases for post-translocation monitoring cost. Translocation and post-translocation monitoring account for 1.20% (median value calculated on 37 files; 1st quartile = 0.29%; 3rd quartile = 4.57%) and 3.12% (median value calculated on 25 files; 1st quartile = 0.56%; 3rd quartile = 13.4%) of the cost of the mitigation hierarchy. The median cost for a translocation operation was 3600 € (1st quartile = 2000 €; 3rd quartile = 7400 € for 40 files). The median cost of post-translocation monitoring for one year was 5400 € (1st quartile = 3300 €; 3rd quartile = 12,000 € for 19 files).

**Fig. 1:**
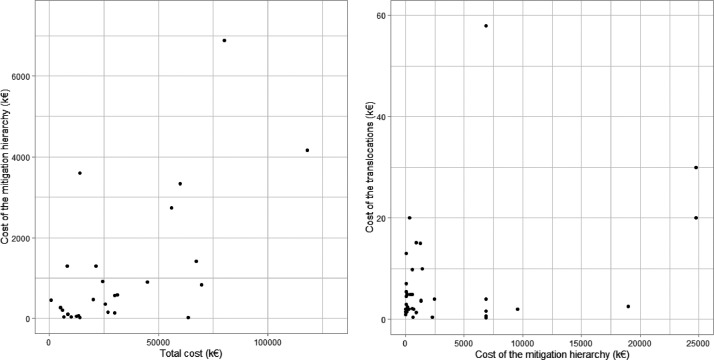
Left, cost of mitigation hierarchy as a function of the total project cost. Right, cost of a translocation operation as a function of the mitigation hierarchy. One outlier point was removed from both graphs.

Fig. 1 shows the relationship between mitigation hierarchy cost and total cost, and mitigation hierarchy cost and translocation operation cost. With a Spearman correlation test, we determined that there was indeed a positive relationship between mitigation hierarchy cost and total project cost (rho=0.59; *p*-value=2e10^−10^). On the other hand, there was no correlation between mitigation hierarchy and translocation operation cost (rho=0.09; *p*-value=0.54). We can explain this by the fact that translocation is a measure among others in the mitigation hierarchy, and translocation is not going to be of greater magnitude when there is a greater number of measures.

## 2 Experimental Design, Materials and Methods

We read 95 derogation files examined by the National Council for the Protection of Nature (in French: “Conseil National de la Protection de la Nature”, hereafter CNPN) between 2018 and 2020 to assess the quality of plant translocation protocols. The CNPN is a French committee, giving an advisory technical and scientific opinion on the consideration of the environment in land planning files. Therefore, these derogation files contain an analysis of the impacts that the project will have on the environment and the measures that will be implemented to mitigate and compensate for these impacts. We analysed suggested protocols for translocating plant populations impacted by development projects, which was the subject of Julien et al. [1]. At the same time, we noted the estimated costs of these same translocation operations. This information is often difficult to access in the literature. The mitigation hierarchy and the total project costs are also available in some of these files. They indicate how well the environment is considered in land planning.

Data were acquired by reading the 95 derogation files. We performed some analyses using R 3.6.3 and R studio with these data. The cost data did not follow a normal distribution (graphically), so we calculated the median percentage that the mitigation hierarchy represented in the total project cost. Then, we estimated the median percentage that translocation and post-translocation monitoring represented in the mitigation hierarchy cost. Finally, we evaluated the median cost of a translocation operation and post-translocation monitoring.

Spearman correlation tests were performed to determine if there is a relationship between mitigation hierarchy cost and total project cost, and between mitigation hierarchy cost and translocation operation cost.

## Ethics Statement

The authors declare that this submission follows the ethical requirements for publication in Data in Brief.

## CRediT authorship contribution statement

**Margaux Julien:** Methodology, Investigation, Formal analysis, Writing – review & editing. **Bruno Colas:** Validation, Supervision. **Serge Muller:** Resources, Validation. **Bertrand Schatz:** Methodology, Validation, Supervision.

## Declaration of Competing Interest

The authors declare that they have no known competing financial interests or personal relationships that could have influenced the work reported in this paper.
